# Incidence Density and Predictors of Multidrug-Resistant Tuberculosis Among Individuals With Previous Tuberculosis History: A 15-Year Retrospective Cohort Study

**DOI:** 10.3389/fpubh.2021.644347

**Published:** 2021-05-28

**Authors:** Qinglin Cheng, Li Xie, Le Wang, Min Lu, Qingchun Li, Yifei Wu, Yinyan Huang, Qingjun Jia, Gang Zhao

**Affiliations:** ^1^Hangzhou Center for Disease Control and Prevention, Hangzhou, China; ^2^School of Medicine, Hangzhou Normal University, Hangzhou, China

**Keywords:** multidrug-resistant, previous tuberculosis, incidence density, predictors, surveillance

## Abstract

**Background:** To date, too little attention has been paid to monitoring and estimating the risk of incident multidrug-resistant tuberculosis (MDR-TB) among individuals with a previous tuberculosis history (PTBH). The purpose of this study was to assess the incidence of and risk factors for MDR-TB in those individuals.

**Methods:** Between 2005 and 2020, a large, retrospective, population-based cohort study was performed in Hangzhou, China. A multivariable Cox regression model was used to evaluate independent predictors of incident MDR-TB among individuals with PTBH.

**Results:** The incidence density of MDR-TB was 22.6 per 1,000 person-years (95% confidence level and an interval of 20.9–24.3) for individuals with PTBH. The incidence of MDR-TB increased significantly in individuals who

• were under 60 years old.

• were male.

• had a history of direct contact.

• came from low-income families.

• worked in high-risk occupations.

• lived in rural areas.

• had a retreatment TB history.

• had an unfavorable outcome in their previous treatment (*P* < 0.05).

In addition, we found that the following factors were significantly linked to the MDR-TB risk among individuals with PTBH (*P* < 0.05):

• sociodemographic factors such as the 21–30 and 31–40 year age groups, or a history of direct contact.

• clinical factors like passive modes of TB case finding (PMTCF), human immunodeficiency virus infection, unfavorable treatment outcomes, retreated TB history, non-standardized treatment regimens of retreatment TB patients, and duration of pulmonary cavities (DPC).

• microbiological factors, such as duration of positive sputum culture.

We also found that the 21–30 year age group, low family income, and PMTCF were significantly linked to incident MDR-TB only in males with PTBH, whilst the 41–50 year age group, extended treatment course, and DPC were significantly associated with female MDR-TB only.

**Conclusion:** The incidence of MDR-TB was high, with a higher rate among subjects with a history of direct contact and unfavorable treatment outcomes. There was a gender difference in the incidence density and risk factors of MDR-TB among individuals with PTBH. Long-term monitoring and gender-specific risk-factor modifications should be given to individuals with PTBH.

## Introduction

Multidrug-resistant tuberculosis (MDR-TB) is associated with severe and fatal respiratory disease in humans. To date, MDR-TB continues to be a serious global public health issue (1). Although the prevalence of MDR-TB in China has increased dramatically in the past decades (2), prevention and early detection of MDR-TB are still inadequate (3).

Continuous monitoring indicates that some MDR-TB patients have a previous history of tuberculosis (PTBH) (e.g., a case after completing TB treatment happens MDR-TB) (4, 5). The most recent MDR-TB epidemic revealed an increased risk of morbidity in individuals with PTBH (6, 7). In our investigation of MDR-TB, we found that the surveillance and management of MDR-TB were less sensitive (i.e., no strategies for early detection) among individuals with PTBH.

Our literature review concluded that a clear understanding of how previous TB cases contributed to future MDR-TB onset was still lacking. Previous reports focused primarily on the prevalence and predictors of MDR-TB in TB patients (5, 8–10). Few researchers have studied the incidence and risk factors of MDR-TB among individuals with PTBH (10–12).

To reduce the morbidity and mortality of MDR-TB, the government and research institutions must address the potential disease burden and multifactor pathogenesis of MDR-TB among individuals with PTBH. We conducted an extensive, retrospective, population-based cohort study to determine the association between PTBH and incident MDR-TB in Hangzhou City, China, between October 1, 2005, and September 30, 2020. The purpose of this study was to (1) assess the incidence density of MDR-TB among individuals with PTBH, and (2) define specific risk factors for MDR-TB in this population.

## Materials and Methods

### Study Design and Settings

The study was conducted in Hangzhou, China. The study cohort included individuals with PTBH, including newly diagnosed TB history (NDTH) and retreated TB history (RTH) with drug-resistance test (DRT) who were recruited in the study between October 1, 2005, and September 30, 2020. For this study, we selected MDR-TB cases from all TB designated hospitals in Hangzhou City. MTB-TB cases were diagnosed by clinicians through Gene Xpert (GX) and traditional drug susceptibility testing (DST) (13).

Subjects were divided into incident MDR-TB group (i.e., the exposure group) and non-incident MDR-TB group (such as the control group) based on DRT results for participants during the follow-up period. Subjects were selected if they

- had a history of TB but did not have MDR-TB during previous treatment episodes;
- were surviving during the study;
- had a history of TB treatment;
- had a confirmed treatment outcome; and
- were available for follow-up.

Subjects were excluded if

**Figure 1:**
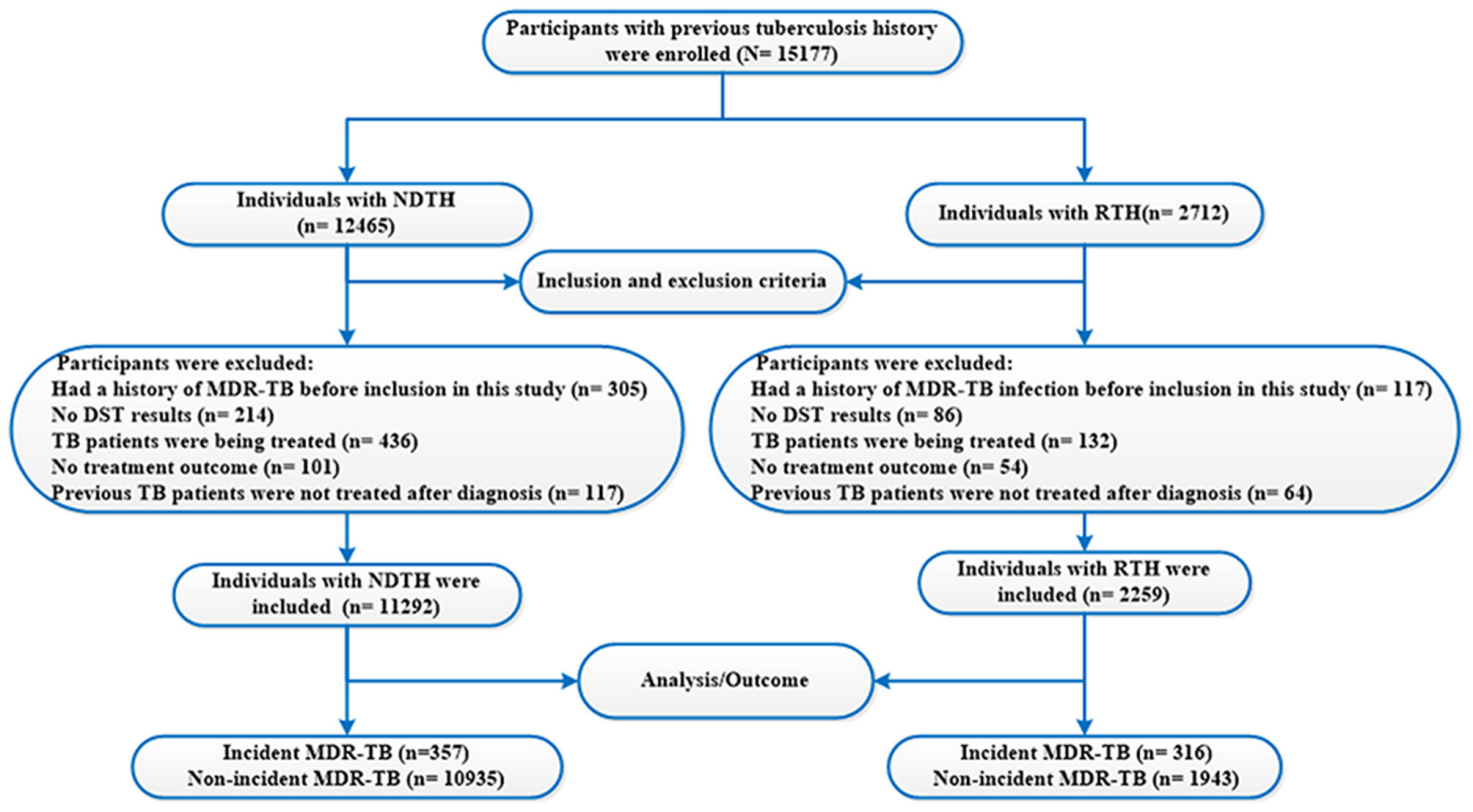
Flow chart of the study population. TB, tuberculosis; MDR-TB, multidrug-resistant tuberculosis; NDTH, newly diagnosed tuberculosis history; RTH, retreated tuberculosis history; DST, drug susceptibility testing.

- they had a history of MDR-TB infection before the previous TB treatment episodes;
- no DST results were reported;
- they were a TB patient being treated (i.e., a case with an anti-TB drug therapy during the course of study);
- no treatment outcome could be identified;
- they were not treated (i.e., a subject who refused treatment after TB diagnosis) (Figure 1).

The starting date of our study referred to the starting date for previous anti-TB treatment. A patient's observation ending date was the end of the incident MDR-TB or September 30, 2020. Incident MDR-TB data for all years were collected between October 1, 2005, and September 30, 2020. Participants were observed until incident MDR-TB was recorded or until September 30, 2020. Treatment regimens (TRs) were formulated based on the treatment history of the TB cases.

### Sample Size Calculation

To calculate the sample size of cohort study, we used the following formula (14):

$$\begin{array}{l} N=[Z_{\alpha }\sqrt{\left(1+\frac{1}{m}\right)\bar{p\;}\;\left(1-\bar{p}\right)}\\ {+Z_{\beta \;}\sqrt{p_{0}\frac{\left(1-p_{0}\right)}{m}+p_{1\;}\left(1-p_{1}\right)}\;]}^{2}/{\left(p_{0}-p_{1}\right)}^{2}\\ \bar{p}=\left(p_{1}+mp_{0}\right)/\left(1+m\right) \end{array}$$

where *N* = sample size; α = alpha (expected significant level, two-tailed test); β = 1– power (expected power, two-tailed test); *Z* statistics (*Z*) —*Z* statistics for confidence level; *Z*_α_ = standard normal variate for level of significance; *Z*_β_ = standard normal variate for power or type 2 error as explained in earlier section; *m* is the number of control subjects per experimental subject; *p*_0_ is the probability of event in controls (*p*_0_ can be estimated as the population prevalence of the event under investigation); *p*_1_ *is* the probability of an event in experimental subjects.

In this study, the investigators presented their results with a 95% confidence interval (CI), *Z*_0.05_ = 1.96 (α = 0.05), *Z*_0.10_ = 1.64 (β = 0.10), *m* = 1, *p*_0_ = 0.0021% (15), and *p*_1_ = 8.0% (16). According to the sample size calculation, our study had to take at least 153 samples in each group. However, subjects were recruited in this study using the “all-comers” principle (17), provided they met the inclusion and exclusion criteria. This approach meant the sample was more likely to be representative of the general population in Hangzhou.

### Data Collection

Data was collected from 10 TB hospitals that provided TB treatment and management in Hangzhou. They included the First Affiliated Hospital, Zhejiang University School of Medicine, Hangzhou Red Cross Hospital, Hangzhou Second People's Hospital, Xiaoshan District People's Hospital, Yuhang District People's Hospital, Lin'an District People's Hospital, Fuyang District People's Hospital, Tonglu County People's Hospital, Chun'an County People's Hospital, and Jiande Second People's Hospital. The database was compiled from existing electronic medical records, a self-designed standard questionnaire, and the National TB Surveillance System (NTSS). The questionnaire was used to collect patients' sociodemographic data. The NTSS was established in 2005 and used to collect patients' clinical and laboratory test data. Sociodemographic data included age, gender, areas of residence, history of direct contact MDR-TB, nationality, family income, occupational risk, education levels, and registered household. Clinical data included modes of TB case-finding, human immunodeficiency virus (HIV) infection, TB patients with severe infection, comorbidities, different PTBH (such as NDTH and RTH), modes of TB case management, treatment outcomes, treatment course, TRs, and chest radiological findings. Laboratory test data included sputum smear, culture, and DST during the baseline and follow-up visits.

All data was collected from the NTSS and entered into an electronic database. Standard participant reporting included the collection by trained investigators of sociodemographic, clinical, and microbiological information, along with initial and follow-up visits.

### Variables and Definitions

In the present study, case definitions and classifications were consistent with the World Health Organization's (WHO) revised TB definitions and reporting framework (18). Incident MDR-TB or non-incident MDR-TB was defined as the main outcome variable. Table 1 shows the definitions used in the study. Based on WHO and national guidelines, we defined and classified the primary covariate variables, and sputum smear, culture, and DST results (18–20).

**Table 1:** Definitions of this study.

**Variables**	**Definitions**
MDR-TB case	A patient infected with TB resistant to at least H and R.
Incident MDR-TB	An MDR-TB patient is diagnosed between the initiating of previous anti-TB treatment and the date of study end.
Non-incident MDR-TB	Incident MDR-TB has not occurred between the initiating of previous anti-TB treatment and the date of study end.
Low-income level	The annual household income is below RMB 150,000 Yuan.
Middle level and above income	The annual household income is greater than or equal to RMB 150,000 Yuan.
High-risk occupation	Such as factory workers, unemployed persons, rural migrant workers.
Low-risk occupation	Such as peasants, teachers, students, service workers, nursing workers, attendants, doctors, fisherfolk, drivers, office workers, and retirees.
Active modes of TB case finding	The suspected or confirmed patients are found according to early clinical symptoms, initiative care-seeking, and high-risk population screening.
Passive modes of TB case finding	The suspected or confirmed patients are found according to physical health examination, differential diagnosis of other diseases, and the screening of close contacts.
A history of direct contact	There is a history of close contact with MDR-TB cases within the past 12 months before the onset of MDR-TB.
Previously treated TB patients	It is defined as patients who have a previous history of treatment with anti-TB drugs for 1 month or more. It includes relapse patients, treatment after failure patients, treatment after loss to follow-up patients, and other previously treated patients.
Retreated TB patients	Refers to patients with initial treatment failure (e.g., NDTPs with sputum positive are still sputum-test-positive at the end of the 5th month or after a course of treatment), relapse cases (e.g., TB has a relapse for the cured NDTPs or NDTPs with completing treatment), returned cases (e.g., re-entry after abandoning therapy), chronic cases, and other (i.e., loss to follow-up, discontinued treatment, and unknown or undocumented therapy outcomes) cases.
TB patients were not treated	Refers to refusing treatment after diagnosis.
Favorable treatment outcome	The standard definition is as follows: (1) TB cases of previous sputum positive are cured after completing treatment; (2) TB cases of previous sputum negative have completed TB treatment, who have presented a remarkable improvement of clinical manifestation.
Unfavorable treatment outcome	Here it refers to previous TB patients who are not cured or have not a remarkable improvement of clinical manifestation after completing treatment, or have not completed TB therapy (i.e., TB cases with discontinued treatment).
Individuals with PTBH	It is defined as follows: (a) subjects have a history of anti-TB treatment (such as a history of newly diagnosed TB treatment or a history of retreated TB treatment) before their inclusion in this study; (b) after a period of anti-TB treatment, a patient is represented as a favorable treatment outcome or an unfavorable treatment outcome.
Different PTBH	Including newly diagnosed TB history and retreated TB history.
2HRZE/4HR	NDTPs are treated by using first-line drug treatment (i.e., R, H, E, and Z) during the intensive treatment phase of 2 months, and using R and H during a 4-month consolidation period; the frequency of TB therapy is once a day.
2H3R3Z3/4H3R3	NDTPs are treated by using first-line drug treatment (i.e., R, H, and Z) during the intensive treatment phase of 2 months, and using R and H during a 4-month consolidation period; the frequency of TB therapy is once every 3 days.
2H3R3Z3E3/4H3R3	NDTPs are treated by using first-line drug treatment (i.e., R, H, E, and Z) during the intensive treatment phase of 2 months, and using R and H during a 4-month consolidation period; the frequency of TB therapy is once every 3 days.
2HREZ/4H3R3	NDTPs are treated by using first-line drug treatment (i.e., R, H, E, and Z) during the intensive treatment phase of 2 months, and using R and H during a 4-month consolidation period; the treatment frequency of NDTPs is once a day and once every 3 days during the intensive and consolidation treatment phases, respectively.
2HRZES/6HRE	RTPs are treated by using TRs (i.e., R, H, E, Z, and S) during the intensive treatment phase of 2 months, and using H, R, and E during a 6-month consolidation period; the frequency of TB therapy is once a day.
3HRZE/6HRE	RTPs are treated by using TRs (i.e., R, H, E, and Z) during the intensive treatment phase of 3 months, and using H, R, and E during a 6-month consolidation period; the frequency of TB therapy is once a day.
3HRZES/6HRE	RTPs are treated by using TRs (i.e., R, H, E, Z, and S) during the intensive treatment phase of 3 months, and using H, R, and E during a 6-month consolidation period; the frequency of TB therapy is once a day.
2H3R3Z3E3S3/6H3R3E3	RTPs are treated by using TRs (i.e., R, H, E, Z and S) during the intensive treatment phase of 2 months, and using H, R, and E during a 6-month consolidation period; the frequency of TB therapy is once every 3 days.
Individualized TRs	According to the clinical experience of doctors, it is defined as the TRs of 4–6 anti-TB drugs (such as 3 or 4 first-line drugs treatment, an injectable second-line drug, and/or a fluoroquinolone).
TRs of NDTPs	Including standardized (i.e., 2HRZE/4HR) and non-standardized (i.e., 2H3R3Z3/4H3R3, 2H3R3Z3E3/4H3R3, 2HREZ/4H3R3, and individualized TRs) TRs.
TRs of RTPs	Including standardized (i.e., 2HRZES/6HRE) and non-standardized (i.e., 2H3R3Z3E3S3/6H3R3E3, 3HRZE/6HRE, and individualized TRs) TRs.
Extended treatment course	Refers to greater than a 6-month course of treatment for NDTPs or greater than a 9-month course of treatment for RTPs.
Standardized treatment course	Refers to a 6-month course of NDTPs treatment and the 8-month treatment course or the 9-month treatment course for RTPs.

*MDR-TB, multidrug-resistant tuberculosis; TB, tuberculosis; PTBH, previous tuberculosis history; NDTPs, newly diagnosed TB patients; RTPs, retreated TB patients; H, isoniazid; R, rifampicin; Z, pyrazinamide; E, ethambutol; S, streptomycin; WHO, World Health Organization; TRs, treatment regimens*.

### Incidence Density

The incidence density of MDR-TB per 1,000 person-years (PYs) was calculated as the number of cases who developed new onset of MDR-TB during the follow-up period divided by the PYs. The PYs were evaluated by using an approximate algorithm [i.e., PYs = (the annual average number of individuals with PTBH) × (total observation years); the annual average number of individuals with PTBH referred to the meaning of individuals with PTBH at the beginning of 2 years in a row] (21). The incidence density was presented with 95% CI using the Poisson distribution. The densities were compared by calculating incidence density ratios with a 95% CI.

### Laboratory Methods

The present study mainly used the methods of traditional detection (e.g., sputum smear and culture) for diagnosing TB cases from 2005 to 2014 (22). Furthermore, the TB diagnosis mainly included molecular biological (i.e., a GX method) and traditional detection methods between 2015 and 2020 (13). Based on the DST result, an MDR-TB case was diagnosed in the designated laboratory. The DST was performed on all culture-positive isolates against first-line (isoniazid, rifampicin, pyrazinamide, ethambutol, and streptomycin) and second-line anti-TB drugs (such as kanamycin and ofloxacin) (20). The methods included conventional DST and GX Mycobacterium TB (MTB)/rifampicin. According to standard procedures, a solid or automated liquid culture media system (BACTEC MGIT 960; Becton Dickinson, Sparks, Maryland, USA) was used for the DST (20). The GX MTB/rifampicin (Cepheid, Sunnyvale, USA) was a semi-nested polymerase chain reaction system with fully automated real time, based on molecular beacon technology (23).

The DST was conducted during follow-up. The detections of conventional DST and GX MTB/rifampicin of collected samples were performed at the TB Program Laboratory of Hangzhou Center for Disease Control and Prevention (a Biosafety Level 3 laboratory with proficiency testing approved by the National Reference Laboratory in China). We excluded MDR-TB cases with laboratory cross-contamination. Borderline TB drugs resistance was considered resistant.

### Statistical Analysis

In this study, a binary variable including incident MDR-TB and non-incident MDR-TB categories was defined as the outcome variable. The analysis of subjects' characteristics was conducted by using the descriptive method. Continuous variables were described using the mean with standard deviation (SD) while categorical data were analyzed using percentage (proportion). Markov chain Monte Carlo (MCMC) method of multiple imputation (MI) was used to impute censored missing data in this study (24). The data was missing at random in this study. The missing-data mechanism was called missing at random according to the probability that a value was missing did not depend on the missing value but did depend on observed quantities (values of variables that were measured). The following variables were used in our MI: family income, comorbidities, treatment course, and duration of negative sputum smear. The MI procedure in R software was used to perform 200 imputations of each variable by using a MCMC approach. We evaluated the initial estimates for MCMC through the expectation-maximization algorithm.

Univariable and multivariable Cox proportional hazards regression models were used to analyze the risk of incident MDR-TB among individuals with PTBH. Subjects who could not be evaluated during the study were excluded from the analysis. A univariable Cox regression analysis was conducted to identify the factors associated with incident MDR-TB. Variables were analyzed using hazard ratio (HR) generated using the same Cox regression analysis.

Subsequently, independent predictors associated with incident MDR-TB were evaluated using HR generated by a multivariable Cox regression model. All variables with a *P* < 0.05 were included the Cox analysis using the backward stepwise method based on the minimum statistics of the Akaike information criterion. Variables with a *P* < 0.05 were considered statistically significant in the Cox analysis and included in the final Cox proportional hazards regression model.

R software (version i 386 4.0.4; www.R-project.org, 2021) was used for all statistical analyses.

## Results

### Characteristics of the Subjects

From 2005 to 2020, a total of 96,573 TB patients were diagnosed in Hangzhou. Of the 96,573 TB patients, 81,396 were excluded (i.e., no DRT result) because the high-risk group of drug-resistant TB (such as TB patients with sputum smear–positive or unsuccessful treatment) was only asked to do the DRT (e.g., the DST of MTB) in China. Finally, 15,177 subjects were enrolled in this study. A flow diagram summarizing the identified eligible subjects is shown in Figure 1. The basic characteristics of the subjects are listed in Table 2.

**Table 2:** Basic characteristics of the study population (*N* = 13,551).

**Variables**	**Incident MDR-TB group [*n* = 673, Mean ± SD or *n* (%)]**	**Non-incident MDR-TB group [*n* = 12,878, Mean ± SD or *n* (%)]**	***P-*value**
**Age (years)**	43.08 ±18.50	51.99 ± 20.14	<0.001^*^
**Gender**			
Male	493 (73.25)	8,719 (67.70)	0.003^*^
Female	180 (26.75)	4,159 (32.30)	
**Nationality**			
Han	665 (98.81)	12,679 (98.45)	0.462
National minority	8 (1.19)	199 (1.55)	
**Occupational risk**			
High-risk	204 (30.31)	2,314 (17.97)	<0.001^*^
Low-risk	469 (69.69)	10,564 (82.03)	
**Education levels**			
High school and below	526 (78.16)	9,357 (72.66)	0.002^*^
Universities and higher	147 (21.84)	3,521 (27.34)	
**Residences**			
Rural areas	253 (37.59)	3,724 (28.92)	<0.001^*^
Urban areas	420 (62.41)	9,154 (71.08)	
**Registered household**			
Migrant individuals with PTBH	277 (41.16)	5,867 (45.56)	0.025^*^
Resident individuals with PTBH	396 (58.84)	7,011 (54.44)	
**Family income**			
Low level	463 (68.80)	7,947 (61.71)	<0.001^*^
Middle level and above	210 (31.20)	4,931 (38.29)	
**Types of MDR-TB diagnosis**			
Traditional susceptibility test	479 (71.17)	8,915 (69.23)	0.286
Gene Xpert MTB/rifampicin	194 (28.83)	3,963 (30.77)	
**Different PTBH**			
NDTH	506 (75.19)	10,786 (83.76)	<0.001^*^
RTH	167 (24.81)	2,092 (16.24)	

* *Statistically significant. MDR-TB, multidrug-resistant tuberculosis; PTBH, previous tuberculosis history; NDTH, newly diagnosed TB history; RTH, retreated TB history; SD, standard deviation; MTB, mycobacterium tuberculosis*.

Of the 13,551 subjects with PTBH, including 12,172 subjects who completed the follow-up study, 89 deaths, the loss to follow-up of 1,004 subjects, and 286 subjects with missing data, were included in the analytic cohort. Of the total, the vast majority was of the Han nationality [13,344 (98.47%)]. Most of the subjects had educational levels of high school and below [9,872 (72.85%)] and low family incomes [8,827 (65.14%)]. The mean age in the study population was 50.96 ± 21.03, and the ratio of males to females was 2.25–1. The main reason for this was that TB occurred mainly in the elderly population or males in China (25).

### Incidence Density of MDR-TB Among Individuals With PTBH

The subjects were observed for a period of 15 PYs in total. Among the 13,551 individuals without MDR-TB at the baseline, there were 673 new cases of MDR-TB. As shown in Table 3, the incidence density of MDR-TB was 22.6 per 1,000 PYs (95% CI 20.9–24.3) among individuals with PTBH in Hangzhou.

**Table 3:** Incidence density of MDR-TB among individuals with PTBH in Hangzhou, China (*N* = 13,551).

**Variables**	**No. of persons**	**Person- years**	**No. of cases**	Incidence density^*^ (95% CI)	**Crude relative risk (95% CI)**
**Age-group**					
<60 years	8,236	17295.6	518	30.0 (27.5–32.6)	2.38 (1.88–3.00)^**^
≥60 years	5,315	12509.1	155	12.4 (10.5–14.5)	1.00
Crude	13,551	29812.2	673	22.6 (20.9–24.3)	
**Gender**					
Male	9,212	21187.6	493	23.2 (21.3–25.4)	1.28 (1.03–1.60)^**^
Female	4,339	9986.7	180	18.0 (15.5–20.8)	1.00
**Nationality**					
Han nationality	13,344	21350.4	665	31.2 (28.9–33.6)	1.22 (0.49–3.01)
National minority	207	314.6	8	25.4 (11.0–49.4)	1.00
**A history of direct contact**					
Yes	709	1772.5	102	57.5 (47.2–69.4)	2.91 (2.20–3.84)^**^
Unknown	3,795	9487.5	182	19.2 (16.5–22.2)	1.00 (0.80–1.26)
No	9,047	20381.2	389	19.1 (17.3–21.1)	1.00
**Family income**					
Low level	8,410	19343.0	463	23.9 (21.8–26.2)	1.34 (1.08–1.66)^**^
Middle level and above	5,141	11837.9	210	17.7 (15.4–20.3)	1.00
**Occupational risk**					
High–risk	2,518	6295.0	204	32.4 (28.2–37.1)	1.88 (1.52–2.33)^**^
Low–risk	11,033	27639.8	469	17.0 (15.5–18.6)	1.00
**Education levels**					
High school and below	9,883	22730.9	526	23.1 (21.2–25.2)	1.27 (0.99–1.61)
Universities and higher	3,668	8076.4	147	18.2 (15.4–21.4)	1.00
**Residences**					
Rural areas	3,977	9544.8	253	26.5 (23.4–29.9)	1.44 (1.17–1.76)^**^
Urban areas	9,574	22977.6	420	18.3 (16.6–20.1)	1.00
**Registered household**					
Migrant individuals with PTBH	6,144	15360.0	277	18.0 (16.0–20.3)	0.91 (0.75–1.11)
Resident individuals with PTBH	7,407	20005.7	396	19.8 (17.9–21.8)	1.00
**Different PTBH**					
RTH	2,259	7864.6	306	38.9 (34.7–43.4)	2.80 (2.30–3.41)^**^
NDTH	11,292	27100.8	367	13.5 (12.2–15.0)	1.00
**Types of MDR–TB diagnosis**					
Traditional susceptibility test	9,394	21606.2	479	22.2 (20.3–24.2)	1.14 (0.92–1.41)
Gene Xpert MTB/rifampicin	4,157	9980.9	194	19.4 (16.8–22.3)	1.00
**Treatment outcome**					
Unfavorable	3,438	7907.4	354	51.1 (46.3–56.2)	4.26 (3.50–5.18)^**^
Favorable	10,113	31363.9	319	8.6 (7.6–9.7)	1.00

* *Per 1000 person-years*.

** *Statistically significant (P < 0.05). PTBH, previous tuberculosis history; MDR-TB, multidrug-resistant tuberculosis; NDTH, newly diagnosed tuberculosis history; RTH, retreated tuberculosis history; CI, confidence interval; MTB, mycobacterium tuberculosis*.

We then examined the incidence density of MDR-TB based on age, gender, nationality, education levels, registered household, family income, occupational risk, residences, history of direct contact, different PTBH, types of MDR-TB diagnosis, and treatment outcomes. Among them, subjects with a history of direct contact had the highest incidence of MDR-TB [57.5 per 1,000 PYs (95% CI 47.2–69.4)]. In contrast, subjects with favorable treatment outcomes had the lowest incidence [8.6 per 1,000 PYs (95% CI 7.6–9.7)]. The incidence of MDR-TB increased significantly for subjects under 60 years of age [crude relative risk 2.38 (95% CI 1.88–3.00)]. It also increased for those with a history of direct contact, male, low family income, high-risk occupation, rural areas, repeated treatment for TB, and unfavorable treatment outcomes (*P* < 0.05) (Table 3).

### Predictors of Incident MDR-TB Among Individuals With PTBH

Table 4 summarizes the results of univariable Cox regression analyses. They focused on the association between an individual covariate and the MDR-TB risk in subjects. Twenty-one of the 38 tested covariates were associated with a high risk of incident MDR-TB in the study population (*P* < 0.05). The significant covariates were:

- sociodemographic characteristics, including the 21–30, 31–40, and 41–50 year age groups, male, a history of direct contact, low family income (LFI), high-risk occupation, high school education and below, residence in rural areas, and migrant individuals with PTBH.
- clinical characteristics, including passive modes of TB case finding (PMTCF), HIV infection, RTH, unfavorable treatment outcome, non-standardized TRs for re-treated TB patients (RTPs), extended treatment course (ETC), and duration of pulmonary cavities (DPC).
- microbiological characteristics, including frequencies of sputum culture, duration of positive sputum culture (DPSC), frequencies of sputum smear, and duration of positive sputum smear (DPSS).

**Table 4:** Univariate Cox regression analysis of risk factors of MDR-TB among individuals with PTBH in Hangzhou, China.

**Variables**	**Total (*****N*** **= 13,551)**		**Stratified by gender**			
			**Male (*****n*** **= 9,212)**		**Female (*****n*** **= 4,339)**	
	***P*-value**	**HR (95% CI)**	***P*-value**	**HR (95% CI)**	***P*-value**	**HR (95% CI)**
**SOCIODEMOGRAPHIC CHARACTERISTICS**						
**Age (years)**						
0–10	0.214	1.57 (0.65–1.86)	0.487	1.12 (0.32–2.25)	0.459	0.94 (0.24–1.91)
11–20	0.253	0.98 (0.38–1.90)	0.156	1.84 (0.36–1.99)	0.105	1.16 (0.34–1.64)
21–30	0.012^*^	2.31 (1.23–2.74)	0.009^*^	1.89 (1.09–2.56)	0.014^*^	2.23 (1.23–4.63)
31–40	<0.001^*^	2.58 (1.47–3.88)	<0.001^*^	2.08 (1.43–4.02)	<0.001^*^	2.64 (1.51–4.97)
41–50	0.032^*^	1.63 (1.11–2.54)	0.045^*^	1.57 (1.07–2.11)	0.014^*^	1.72 (1.09–2.55)
51–60	0.102	1.87 (0.35–2.09)	0.231	1.48 (0.71–2.06)	0.308	1.91 (0.78–2.94)
>60	Reference		Reference		Reference	
**Gender**						
Male	0.021^*^	1.61 (1.12–2.09)	NA	NA	NA	NA
Female	Reference		NA	NA	NA	NA
**Nationality**						
Han	0.457	1.09 (0.89–2.43)	0.435	0.99 (0.71–2.34)	0.326	1.04 (0.88–2.65)
National minority	Reference		Reference		Reference	
**A history of direct contact**						
Yes	<0.001^*^	5.24 (2.02–7.87)	<0.001^*^	3.91 (1.54–5.20)	<0.001^*^	5.64 (1.88–7.63)
Unknown	0.410	1.81 (0.81–1.93)	0.389	1.13 (0.33–1.86)	0.325	1.52 (0.49–2.68)
No	Reference		Reference		Reference	
**Family income**						
Low level	0.037^*^	1.49 (1.12–2.08)	0.022^*^	1.57 (1.09–2.87)	0.035^*^	1.29 (1.07–2.96)
Middle level and above	Reference		Reference		Reference	
**Occupational risk**						
High–risk	0.014^*^	1.79 (1.06–2.24)	<0.001^*^	1.71 (1.13–2.67)	0.105	1.87 (0.61–2.12)
Low–risk	Reference		Reference		Reference	
**Education levels**						
High school and below	0.041^*^	1.26 (1.03–1.85)	0.034^*^	1.28 (1.05–2.11)	0.101	1.69 (0.67–2.98)
Universities and higher	Reference		Reference		Reference	
**Residences**						
Rural areas	0.006^*^	1.72 (1.15–2.23)	<0.001^*^	1.77 (1.18–2.54)	0.099	1.88 (0.67–2.22)
Urban areas	Reference		Reference		Reference	
**Registered household for individuals with PTBH**						
Migrant	0.036^*^	1.45 (1.13–2.08)	0.015^*^	1.62 (1.03–2.35)	0.044^*^	1.29 (1.04–1.91)
Resident	Reference		Reference		Reference	
**CLINICAL CHARACTERISTICS**						
**Modes of TB case finding**						
Passive	<0.001^*^	2.56 (1.59–5.31)	<0.001^*^	3.01 (1.51–6.35)	0.001^*^	2.14 (1.52–5.02)
Active	Reference		Reference		Reference	
**Comorbidities**						
Yes	0.107	1.32 (0.88–1.79)	0.045^*^	1.26 (1.04–2.91)	0.208	0.99 (0.43–1.89)
No	Reference		Reference		Reference	
**HIV infection**						
Positive	<0.001^*^	2.03 (1.22–2.65)	<0.001^*^	2.24 (1.21–3.87)	<0.001^*^	1.97 (1.23–3.91)
Negative	Reference		Reference		Reference	
**TB patients with severe infection**						
Yes	0.452	1.39 (0.55–1.84)	0.320	1.51 (0.35–2.54)	0.324	1.24 (0.56–1.80)
No	Reference		Reference		Reference	
**Modes of TB case management**						
FMM or self–management	0.099	1.37 (0.82–2.35)	0.191	1.57 (0.79–2.44)	0.003^*^	1.42 (1.05–2.11)
CDM	Reference		Reference		Reference	
**Different PTBH**						
RTH	<0.001^*^	2.84 (1.48–3.99)	<0.001^*^	2.88 (1.25–3.57)	<0.001^*^	3.02 (1.67–5.87)
NDTH	Reference		Reference		Reference	
**Treatment outcomes**						
Unfavorable	<0.001^*^	3.98 (1.25–6.45)	<0.001^*^	3.21 (1.59–7.25)	<0.001^*^	4.51 (1.98–7.67)
Favorable	Reference	Reference		Reference	
**TREATMENT REGIMENS**						
**Newly diagnosed TB patients**						
Non–standardized	0.112	1.47 (0.94–3.02)	0.089	1.54 (0.92–3.11)	0.159	1.41 (0.57–2.52)
Standardized	Reference		Reference		Reference	
**Retreated TB patients**						
Non–standardized	<0.001^*^	2.98 (1.76–5.09)	<0.001^*^	2.41 (1.49–4.01)	<0.001^*^	3.68 (1.42–5.36)
Standardized	Reference		Reference		Reference	
**Treatment course, months**						
Extended	<0.001^*^	1.23 (1.02–3.69)	0.029^*^	1.20 (1.03–2.02)	<0.001^*^	1.18 (1.02–2.99)
Standardized	Reference		Reference		Reference	
**Chest radiological findings**						
FCXE	0.248	0.65 (0.39–1.26)	0.345	1.41 (0.61–2.32)	0.201	0.78 (0.25–1.56)
DPC, months	<0.001^*^	1.25 (1.03–2.38)	0.012^*^	1.35 (1.14–2.46)	<0.001^*^	1.23 (1.08–2.78)
DPMT, months	0.587	0.99 (0.23–1.87)	0.423	1.62 (0.67–2.56)	0.138	0.88 (0.42–1.85)
DAIF, months	0.254	1.56 (0.49–1.76)	0.102	1.63 (0.56–2.31)	0.289	1.60 (0.39–2.03)
DWCRF, months	0.369	1.51 (0.41–2.35)	0.298	1.55 (0.44–2.05)	0.463	0.99 (0.56–1.91)
**MICROBIOLOGICAL CHARACTERISTICS**						
FSC	0.002^*^	0.84 (0.62–0.96)	0.001^*^	0.91 (0.25–0.99)	0.012^*^	0.83 (0.44–0.98)
DPSC, months	<0.001^*^	1.49 (1.23–2.69)	<0.001^*^	1.31 (1.08–2.04)	<0.001^*^	1.67 (1.21–2.45)
DNSC, months	0.088	0.56 (0.83–1.95)	0.045^*^	0.89 (0.34–0.98)	0.205	0.76 (0.45–1.94)
DWSC, months	0.230	1.84 (0.46–2.02)	0.367	1.34 (0.43–2.09)	0.334	0.89 (0.43–1.78)
FSS	0.023^*^	0.88 (0.71–0.98)	0.014^*^	0.69 (0.45–0.97)	0.087	0.98 (0.77–1.97)
DPSS, months	<0.001^*^	1.59 (1.08–2.51)	0.003^*^	1.49 (1.11–1.97)	<0.001^*^	1.51 (1.14–2.09)
DNSS, months	0.364	0.56 (0.25–1.18)	0.456	0.76 (0.48–1.99)	0.437	0.81 (0.47–1.79)
DWSS, months	0.217	0.64 (0.25–1.68)	0.317	0.94 (0.33–1.88)	0.348	0.92 (0.24–1.85)

* *Statistically significant. PTBH, previous tuberculosis history; MDR-TB, multidrug-resistant tuberculosis; TB, tuberculosis; HIV, human immunodeficiency virus; NDTH, newly diagnosed TB history; RTH, retreated TB history; HR, hazard ratio; CI, confidence interval; FMM, family members' management; CDM, community doctor management; FCXE, frequencies of chest X-ray examination; DPMT, duration of pulmonary miliary tubercles; DPC, duration of pulmonary cavities; DAIF, duration of abnormal imaging findings; DWCRF, duration without chest radiological findings; FSC, frequencies of sputum culture; DPSC, duration of positive sputum culture; DNSC, duration of negative sputum culture; DWSC, duration without sputum culture; FSS, frequencies of sputum smear; DPSS, duration of positive sputum smear; DNSS, duration of negative sputum smear; DWSS, duration without sputum smear; NA, not available*.

The remaining 17 covariates were not associated with incident MDR-TB among individuals with PTBH (*P* > 0.05):

- three age groups: 0–10, 11–20, and 51–60 years.
- nationality.
- a history of direct contact (e.g., unknown).
- TB patients with severe infection.
- comorbidities.
- family members' management or self-management for TB patients.
- non-standardized TRs for newly diagnosed TB patients.
- frequencies of chest X-ray examination.
- duration of pulmonary miliary tubercles.
- duration of abnormal imaging findings.
- duration without chest radiological findings.
- duration of negative sputum culture.
- duration without sputum culture.
- duration of negative sputum smear.
- duration without sputum smear.

To further explore the independent predictors of incident MDR-TB in individuals with PTBH, we conducted a multivariable Cox regression analysis. Table 5 summarizes the results of the analysis for this population. The Cox analysis demonstrated a significant MDR-TB risk in subjects with PTBH and the following characteristics:

- two age groups: 21–30 and 31–40 years.
- a history of direct contact.
- PMTCF.
- HIV infection.
- RTH.
- unfavorable treatment outcome.
- non-standardized TRs of RTPs.
- DPC.
- DPSC.

**Table 5:** Multivariate Cox regression analysis of risk factors of MDR-TB among individuals with PTBH in Hangzhou, China.

**Variables**	**Total (*****N*** **= 13,551)**		**Stratified by gender**			
			**Male (*****n*** **=9,212)**		**Female (*****n*** **=4,339)**	
	***P*-value**	**HR (95% CI)**	***P*-value**	**HR (95% CI)**	***P*-value**	**HR (95% CI)**
**Sociodemographic characteristics**						
**Age groups (years)**						
21–30	0.035^*^	1.54 (1.09–2.25)	0.030^*^	1.61 (1.09–2.35)	0.101	1.87 (0.90–2.78)
31–40	<0.001^*^	2.00 (1.62–2.47)	<0.001^*^	1.57 (1.23–2.00)	<0.001^*^	2.94 (1.96–4.42)
41–50	0.088	1.99 (0.90–2.55)	0.201	1.80 (0.51–2.59)	0.024^*^	1.67 (1.05–2.47)
Male	0.069	1.45 (0.95–2.28)	NA	NA	NA	NA
A history of direct contact	<0.001^*^	5.04 (1.53–10.22)	0.002^*^	5.42 (1.98–9.97)	<0.001^*^	3.63 (1.67–7.03)
Low family income	0.054	1.24 (0.91–1.54)	0.030^*^	1.32 (1.03–1.70)	0.943	1.01 (0.68–1.50)
High–risk occupation	0.081	1.39 (0.89–1.76)	0.444	1.18 (0.77–1.80)	NA	NA
High school and below	0.107	1.36 (0.68–1.71)	0.201	1.33 (0.48–1.73)	NA	NA
Rural areas	0.153	1.31 (0.56–1.76)	0.233	1.44 (0.73–1.97)	NA	NA
Migrant individuals with PTBH	0.079	1.69 (0.92–2.54)	0.102	1.78 (0.65–2.27)	0.449	1.87 (0.60–3.25)
**Clinical characteristics**						
Passive modes of TB case finding	<0.001^*^	1.98 (1.48–5.05)	0.013^*^	1.91 (1.23–5.58)	0.088	2.02 (0.79–4.10)
Comorbidities	NA	NA	0.235	1.68 (0.45–2.36)	NA	NA
HIV infection	<0.001^*^	1.92 (1.23–2.85)	<0.001^*^	1.99 (1.14–4.06)	0.018^*^	1.86 (1.06–2.87)
Family members' management or self–management for TB cases	NA	NA	NA	NA	0.091	1.94 (0.83–3.68)
Retreated TB history	0.005^*^	2.13 (1.11–3.99)	0.036^*^	1.97 (1.26–3.52)	<0.001^*^	2.21 (1.42–5.15)
Unfavorable treatment outcome	<0.001^*^	3.06 (1.88–5.31)	<0.001^*^	3.04 (1.90–7.12)	<0.001^*^	3.11 (1.91–6.82)
Non–standardized TRs for re–treated TB patients	0.011^*^	2.19 (1.35–3.98)	0.004^*^	2.04 (1.58–3.81)	<0.001^*^	2.27 (1.30–4.05)
Extended treatment course, months	0.092	1.35 (0.86–2.97)	0.109	1.25 (0.78–2.65)	0.001^*^	1.49 (1.07–2.84)
Duration of pulmonary cavities, months	0.015^*^	1.41 (1.05–1.94)	0.215	1.28 (0.54–1.83)	0.009^*^	1.79 (1.14–2.52)
**Microbiological characteristics**						
Frequencies of sputum smear	0.204	0.87 (0.55–1.67)	0.145	0.94 (0.64–1.83)	NA	NA
Duration of positive sputum smear, months	0.114	1.61 (0.79–1.90)	0.201	1.43 (0.61–2.14)	0.098	1.72 (0.89–2.41)
Frequencies of sputum culture	0.314	0.81 (0.24–1.89)	0.213	0.59 (0.21–1.67)	0.287	0.97 (0.54–1.84)
Duration of positive sputum culture, months	<0.001^*^	1.59 (1.15–2.36)	<0.001^*^	1.79 (1.22–2.89)	<0.001^*^	1.44 (1.19–2.27)
Duration of negative sputum culture, months	NA	NA	0.231	0.93 (0.54–1.95)	NA	NA

* *Statistically significant. PTBH, previous tuberculosis history; MDR–TB, multidrug-resistant tuberculosis; TB, tuberculosis; HIV, human immunodeficiency virus; TRs, treatment regimens; HR, hazard ratio; CI, confidence interval; NA, not available*.

From this model, we could also see that a history of direct contact was the most striking predictor for incident MDR-TB in this population (OR 5.04, 95% CI: 1.53–10.22, *P* < 0.001).

To explore possible gender differences in the association between incident MDR-TB and predictors, we performed gender-stratified, multiple regression analyses. Analysis stratified by gender showed that the 21–30 year age group, LFI, and PMTCF were significantly linked to incident MDR-TB only in males, whilst the 41–50-year age group, ETC, and DPC were significantly associated with female MDR-TB only. Seven variables (i.e., the 31–40 year age group, a history of direct contact, HIV infection, RTH, unfavorable treatment outcome, non-standardized TRs of RTPs, and DPSC) were related to incident MDR-TB in both genders (Table 5). The models also indicated that of all of the independent predictors, a history of direct contact was the strongest impact factor for both male MDR-TB (OR 5.42, 95% CI: 1.98–9.97, *P* = 0.002) and female MDR-TB (OR 3.63, 95% CI: 1.67–7.03, *P* < 0.001) (Table 5).

## Discussion

In this study, we conducted a 15-year retrospective cohort study to explore the incidence and risk factors of MDR-TB in individuals with PTBH. Our findings may provide more reliable evidence in developing prevention and control strategies for MDR-TB among individuals with PTBH. Because the pathogenic mechanism of MDR-TB has not been fully clarified, we can only decrease the risk of incident MDR-TB by modifying potential risk factors. Thus, we anticipate these results will be useful in reducing the disease burden of MDR-TB and improving risk monitoring and management of MDR-TB among individuals with PTBH.

### Incidence Density of MDR-TB

The incidence density of MDR-TB reported in this study is high, with 22.6 per 1,000 PYs from individuals with PTBH in Hangzhou, China. This finding broadly supports the work of other studies in this area linking MDR-TB with previously treated patients (26). One implication of this finding is the possibility that the government and TB control officials should immediately take measures to monitor and manage individuals with PTBH. As demonstrated in our study, the incidence of MDR-TB increased significantly in subjects under 60 years old and characterized by a history of direct contact, male, low family income, high-risk occupation, rural area of residence, RTH, and unfavorable treatment outcomes. Up to now, China has experienced significant challenges when facing a high incidence of MDR-TB (1). One possible explanation for this may be that the high-risk MDR-TB population is often not well-monitored and managed. In addition, because the TB prevalence level stays high in China, which can, in part, explain the high incidence of MDR-TB (27). To reduce the burden of MDR-TB, interventions should be initiated for the subjects in those high-risk groups.

In our study, the highest incidence density was found in close contact with the MDR-TB case among individuals with PTBH. A possible explanation for this might be that most subjects did not use personal protective measures when they closely contacted the MDR-TB case (28). That strong association implies that interventions like early isolation and treatment of MDR-TB patients, personal protective measures for susceptible persons, and early detection of close contacts are urgently needed to reduce MDR-TB incidence among individuals with PTBH. It is noteworthy that the incidence of MDR-TB was higher amongst individuals with unfavorable treatment outcomes in their previous treatment episodes. In the current study, comparing NDTH with RTH showed that the incidence of MDR-TB was higher in individuals with RTH. A similar trend was observed in previous studies that evaluated the prevalence of MDR-TB in TB patients (6, 10, 29). Enhanced attention and long-term monitoring should be given to patients who have been treated previously, especially those with a history of direct contact, unfavorable treatment outcomes or RTH.

### Predictors of Incident MDR-TB

To our best knowledge, this is the first study to examine the associations between MDR-TB and a broad range of potential risk factors (i.e., epidemiological, clinical, and bacteriological factors) among a large sample of the Chinese population with PTBH. A notable finding in this study was that 10 independent predictors are associated with the increased risk of MDR-TB among individuals with PTBH. These findings were also reported in many previous studies of TB patients (6, 10, 29).

Notably, this study also observed that RTPs are treated using non-standardized TRs (i.e., 2H3R3Z3E3S3/6H3R3E3, 3HRZE/6HRE, and individualized TRs), which increased the risk of incident MDR-TB dramatically. Thus, it can be seen that the acquired infection due to poor TRs during TB treatment is one of the main risk factors for MDR-TB. This association might be attributed to the increased chance of drug resistance with longer exposure to anti-TB drugs (30). Furthermore, the treatment of non-standardized TRs will likely lead to an increased MDR-TB risk by using substandard anti-TB drugs (i.e., poor drug dosages) in TB cases (31). To decrease the risk of MDR-TB, standardized TRs must be implemented by RTPs. However, in reviewing the literature, we found that there were two distinct standpoints for non-standardized TRs. On the one hand, from a programmatic perspective, one would like to see standardized treatment regimens (32). On the other hand, from a personalized medicine perspective, if proper DST or sequencing is available, personalized regimens might be indicated for specific patients (33). Thus, the selection of TRs for TB patients should be based on scientific and precise assessment.

Surprisingly, the present study found a relationship between the PMTCF (such as physical examination, contact examination, and differential diagnosis of other diseases) and incident MDR-TB. A possible explanation for this association is that the increasing risk of MDR-TB may originate from TB cases who were not diagnosed due to a failure of diagnostics at baseline diagnosis. Further analysis reveals that the main reason for this is that there is inadequate sputum smear and culture, the lack of DST, low sensitivity for detecting TB, and poor patient compliance. This reminds us that adequate medical support (i.e., sputum smear, sputum culture, and DST) should be considered by the government. The detection sensitivity for TB should be strengthened in the medical institution. In addition, a health communication schedule with the knowledge of TB diagnosis and treatment should be provided to increase patient compliance. Another possible reason is that delayed diagnosis and treatment of TB may increase the risk of MDR-TB (34). As far as we know, PMTCF may lead to the delayed finding of TB cases. If the TB case could not be found timely, the diagnosis and treatment of TB would be delayed. Maybe the TB case would develop into serious TB leading to an extended course of treatment, which might become a risk factor associated with MDR-TB (35). This study provides important evidence that active modes of TB case finding are beneficial for preventing or reducing MDR-TB morbidity.

Most importantly, we used a stratified comparison to analyze gender differences in the predictors of MDR-TB that were found in individuals with PTBH. There were many differences in predictors for incident MDR-TB between the sexes. These findings might be attributed to certain gender disparities with sociodemographic, therapeutic and managed factors implicated in the development of MDR-TB (32, 36). In fact, the 21–30 year age group, LFI, and PMTCF in males with PTBH are associated with incident MDR-TB. Several possible reasons for this increased risk are as follows. First, it may be due to the high-intensity of work and study leading to decreased immunity. Second, TB cannot obtain timely diagnosis, therapy, and management because the males usually need to go out to work to support their family (i.e., the floating population). We also found that the 41–50 year age group, ETC, and DPC contributed to female MDR-TB only. Although the underlying mechanisms are unclear, a possible explanation for these results may be the lack of adequate nutrition due to reducing body weight in females with PTBH (37). Women might have a low body mass index (BMI) because of poor nutrition and therefore develop MDR-TB. In addition, they might develop MDR-TB due to anxiety for the BMI and poor health (38). If the anxiety would be further developed, the risk of anorexic might be greatly increased. Subsequently, it would promote the occurrence of MDR-TB. Similarly, these factors might lead to ETC and longer DPC for females in their previous treatment episodes (39). Interestingly, the current study found that the risk of MDR-TB had the difference of age groups between genders. It could therefore be assumed that a health promotion program should be performed in males with the 21–40 years age group and females with the 31–50 years age group for the control and prevention of MDR-TB. In summary, gender-specific intervention programs against MDR-TB among individuals with PTBH should be considered to reduce the MDR-TB risk by modifying risk factors.

Finally, it should be reminded that the present study indicated that the risk of MDR-TB among individuals with PTBH was attributed mainly to acquired infection via exposure to a case of MDR in the household or community. The present finding showed that there was an urgent need to contain the epidemic of MDR-TB through potential intervention strategies (such as early detection, early isolation, early diagnosis, and early anti-TB treatment of MDR-TB case, and personal protective measures of the susceptible population) in individuals with PTBH. Moreover, the government would need to carry out a health education program on the knowledge of TB infection control in the household or community. Although these findings were also reported in many previous studies of TB patients (5–11). For example, unfavorable treatment outcomes remain a primary concern for the control of MDR-TB among individuals with PTBH. To monitor and manage the risk of MDR-TB, the focus must be on high-risk factors in individuals with PTBH.

Our studies had several limitations. First, it was a retrospective study, which might cause recall bias. To reduce recall bias, we collected the first follow-up records [i.e., epidemiological history including clear contact history and unclear contact history, demographic data (such as age, gender, occupation, and areas), and clinical data] after the diagnosis of MDR-TB through the TB information system. Secondly, we failed to include some long-established impact factors (e.g., the frequency or intensity of exposure). Thirdly, there are some potential confounders like the psychological health of TB case, the stability, malabsorption, and quality of TB drug could not be controlled. Fourthly, our study might have a selection bias. For example, we might not include all MDR-TB cases, due to non-access or access at other, non-hospital facilities. To reduce selection bias, we have retrieved and collected medical records of MDR-TB cases from hospitals outside Hangzhou through the NTSS. However, we might not include MDR-TB cases with non-access or non-hospital facilities. According to our investigation, there was little for patients with non-access or non-hospital facilities in China. Fifth, the present study has the limitations of using electronic data (i.e., non-standardized, especially over a long period). Finally, in order to reduce limitations, we performed additional measures such as the multicenter research, follow-up of the participants, and the inclusion of additional predictors. Moreover, we verified the validity and reliability of electronic data by using the face-to-face interview among 100 participants with random selection. Despite several limitations of this study, there are limited reports on impact factors of MDR-TB among individuals with PTBH. We feel these findings would instill some cognition on the novel strategy of prevention and control of incident MDR-TB.

## Conclusion

In conclusion, our study showed that, in individuals with PTBH, the incidence of MDR-TB is high, with higher rates among subjects with a history of direct contact and unfavorable treatment outcome. Enhanced attention and long-term monitoring should be given to patients who have been treated previously, especially those with a history of direct contact or previous unfavorable treatment outcomes. This study broadens our knowledge of MDR-TB as a growing public health issue in China and underscores the necessity of healthcare plans for the prevention of MDR-TB.

We identified four primary risk factors for MDR-TB among individuals with PTBH. They included a history of direct contact, unfavorable treatment outcomes, non-standardized TRs for RTPs, and retreated TB history. We also found that there was a gender difference in risk factors of MDR-TB among individuals with PTBH. Therefore, we offer the following recommendations:

- provide a personal protection program of close contacts for MDR-TB.
- increase the rate of favorable treatment outcomes in TB cases.
- treat TB patients with standardized TRs (especially RTPs).
- strengthen early detection of TB.
- modify gender-specific risk factors.

Such measures are critical to preventing the spread of infection from MDR-TB in individuals with PTBH.

## Data Availability Statement

The original contributions presented in the study are included in the article/supplementary material, further inquiries can be directed to the corresponding author/s.

## Ethics Statement

The studies involving human participants were reviewed and approved by the Ethics Committee of the Hangzhou Center for Disease Control and Prevention. Written informed consent to participate in this study was provided by the participants' legal guardian/next of kin.

## Author Contributions

All authors contributed to the data analysis, prepared the draft report, revised the report, prepared the final report, gave final approval for the report to be published, and agreed to be accountable for all aspects of the study.

## Conflict of Interest

The authors declare that the research was conducted in the absence of any commercial or financial relationships that could be construed as a potential conflict of interest.

## Abbreviations

TB: tuberculosis
MDR-TB: multidrug-resistant tuberculosis
PTBH: previous tuberculosis history
NDTH: newly diagnosed TB history
RTH: retreated TB history
DRT: drug-resistance test
GX: Gene Xpert
DST: drug susceptibility testing
TRs: treatment regimens
CI: confidence interval
NTSS: National TB Surveillance System
HIV: human immunodeficiency virus
WHO: World Health Organization
PYs: person-years
MTB: mycobacterium tuberculosis
DPSC: duration of positive sputum culture
DPSS: duration of positive sputum smear
SD: standard deviation
MCMC: Markov chain Monte Carlo
MI: multiple imputation
HR: hazard ratio
LFI: low family income
RTPs: retreated TB patients
ETC: extended treatment course
DPC: duration of pulmonary cavities
DPSC: duration of positive sputum culture
DPSS: duration of positive sputum smear
PMTCF: passive modes of TB case findings
FCXE: frequencies of chest X-ray examination
DPMT: duration of pulmonary miliary tubercles
FMM: family members' management
CDM: community doctor management
DAIF: duration of abnormal imaging findings
DWCRF: duration without chest radiological findings
FSC: frequencies of sputum culture
DNSC: duration of negative sputum culture
DWSC: duration without sputum culture
FSS: frequencies of sputum smear
DNSS: duration of negative sputum smear
DWSS: duration without sputum smear
NA: not available
BMI: body mass index.
